# Numerical prediction of the optimal shield tunneling strategy for tunnel construction in karst regions

**DOI:** 10.1371/journal.pone.0252733

**Published:** 2021-06-04

**Authors:** Zhen Liu, Weihua Ming, Jieming Li, Cuiying Zhou, Lihai Zhang

**Affiliations:** 1 School of Civil Engineering, Sun Yat-sen University, Guangzhou, China; 2 Guangdong Engineering Research Centre for Major Infrastructure Safety, Sun Yat-sen University, Guangzhou, China; 3 Department of Infrastructure Engineering, University of Melbourne, Melbourne, Australia; China University of Mining and Technology, CHINA

## Abstract

Shield tunneling in karst areas poses significant challenges, as vibration caused by the shield machine can disturb the stability of the karst caves, ultimately resulting in the collapse of a tunnel. In the present study, a numerical model involving an iteration process was developed based on the Mindlin solution scheme to identify the optimal shield tunneling speed for minimizing the disturbance to karst cave stability. The developed model was then implemented to investigate an underground tunnel constructed in a karst region with different shield tunneling strategies. By using the variation in the energy density of a karst cave as a performance index, the model predicts that when approaching the affected zone of a karst carve (*e*.*g*., approximately 5 m from the carve), the shield tunneling machine should be controlled within a certain speed (*i*.*e*., < 30 mm/min). Once the shield tunneling machine moves into the affected zone of the cave, the speed of the machine needs to be decelerated to 11 mm/min, and the speed of 30 mm/min can be restored when the shield machine moves out of the affected zone. This finding demonstrates that the developed model could potentially be used to identify the optimal shield tunneling speed to minimize the disturbance to karst cave stability and ensure the safety of tunnel construction in karst regions.

## 1 Introduction

With rapid urbanization worldwide, large-scale tunnel construction for underground transportation has become increasingly popular. The shield tunneling method is one of the main tunnel construction methods due to its high efficiency and low disturbance to the surrounding rock [1, 2]. Karst landforms account for approximately 12% of the global land area [3] and 1/3 of the land area of China [4]. Therefore, shield tunneling construction in karst areas is inevitable with continued urbanization [5, 6]. When passing a karst cave during shield tunneling, the vibration caused by the shield machine could affect the stability of the cave, ultimately leading to the collapse of the tunnel [7–10] and resulting in construction delays, cost overruns, and even loss of life [11–13].

Current strategies for ensuring the stability of karst caves during shield tunneling include maintaining a safe distance between the shield driving route and karst caves [14, 15]. However, this approach could lead to an increase in construction costs, and the shield driving route sometimes cannot be changed in engineering practice. Thus, to minimize the disturbance to the adjacent karst caves, it is necessary to develop new shield tunneling strategies by controlling the shield tunneling speed by adjusting the pressure of the earth bin, the thrust of the jack, the torque of the cutter and the rotation speed [16, 17].

Previous theoretical studies on shield tunneling mainly focused on numerical modeling and empirical model development. In terms of numerical simulation, Zhang used numerical analyse to estimate the surface displacement along the tunnel axis and the surface-zone affected by the shield tunneling [18]. Li established a mathematical model of the shield tunnelling speed by using multiple nonlinear regression and optimized the shield parameters by considering the properties of composite strata [19]. In addition, the correlation between different shield tunneling speeds and their impact on the stability of surrounding structures was recently numerically simulated [20, 21]. However, the goal of accurately simulating tunnel construction in karst regions has not been fully achieved. In addition, due to the complex nature of boundary conditions, numerical simulation processes are normally computationally expensive. To overcome the difficulties encountered in numerical simulation, some empirical approaches have been developed to estimate the optimal shield tunneling speed [22, 23]. However, these empirical models have limited capabilities in considering the influence of nearby karst caves during shield tunneling. Therefore, the purpose of this study is to develop a new numerical model that can identify the optimal shield tunneling speed to ensure the safety of underground tunneling projects constructed in a karst region.

In the present study, a numerical model was developed based on the Mindlin solution scheme to identify the optimal shield tunneling speed that results in minimal disturbance to the stability of an adjacent karst cave. The performance index of the effect of shield tunneling speed on the stability of a karst cave was defined as the variation in energy density at the top of the karst cave. The developed model was validated using an engineering case study. This paper provides a method for optimal control of the shield tunneling speed considering the disturbance to the adjacent karst caves.

## 2 Methods

### 2.1 Modeling a shield machine passing a karst cave

**Fig 1:**
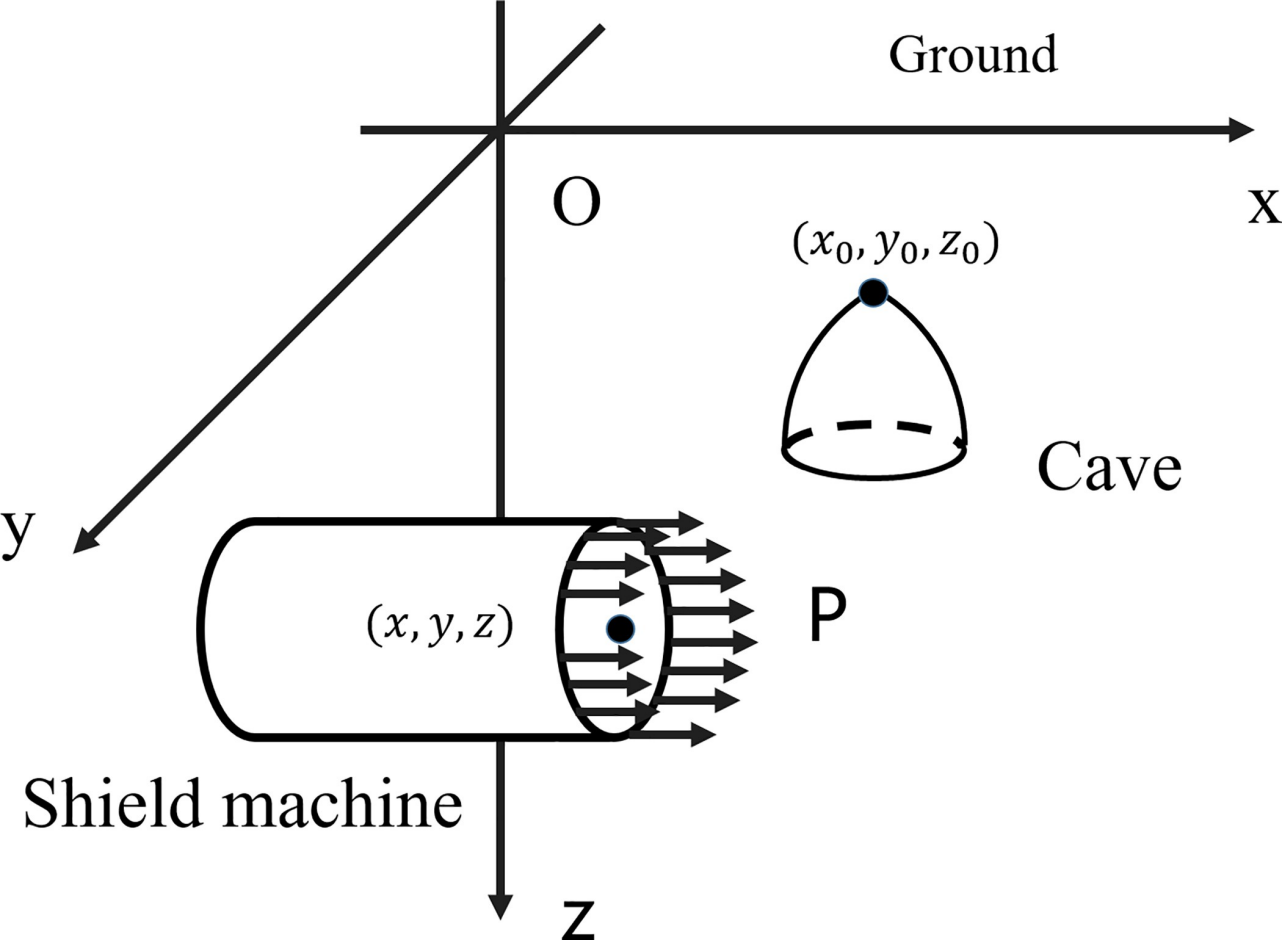
Schematic diagram showing a shield machine passing a nearby karst cave.

As shown in Fig 1, a shield machine passes a karst cave. To simplify the complex modeling problem, the following assumptions are made in this study:

- The complex geotechnical formation can be treated as an elastic half space [24–27];
- The rock and soil mass are modeled as a homogeneous elastic semi-infinite medium;
- The shield tunneling machine bores along a straight horizontal line in the rock;
- The front thrust force of the shield machine generates a uniformly distributed loading on the circular excavation surface;
- The influences of shield friction and synchronous grouting are ignored.

Analytical elasticity solutions provide an efficient means of performing a first approximate analysis in foundation engineering. Engineering practice has proven that a homogeneous elastic semi-infinite medium can be used to model rock and soil to meet the general engineering requirements [25–27]. In this paper, the Mindlin solution is used to study the deformation in shield tunneling [28, 29].

The movement of the shield tunneling machine can be described by the change in the coordinates of the center of the excavation face center with time, *i*.*e*., (*x*(*t*),*y*,*z*). Thus, the shield tunneling speed *u*(*t*) can be obtained:

$$\dot{x}=u(t)$$ (1)

In practice, *u*(*t*) needs to satisfy the constraint condition. The allowable shield tunneling speed (*U*_*ad*_) can be defined as *v*_*min*_<*u*<*v*_*max*_. Therefore, the permissible control set is defined as

$$U_{ad}=\{u(t)|v_{min}<u(t)<v_{max}\}$$ (2)

*v*_*min*_ is the minimum shield tunneling speed, and *v*_*max*_ is the maximum speed.

The stability of a karst cave could be disturbed when the shield tunneling machine is working within a certain range from the karst cave [30, 31]. Given that the radius of the disturbance range is *R*_*d*_ and the coordinate of the cave roof is (*x*_0_, *y*_0_, *z*_0_), we can obtain

$$x(t_{0})=x_{0}-R_{d}$$ (3)

$$x(t_{1})=x_{0}+R_{d}$$ (4)

where *t*_*0*_ is the time when the shield tunneling machine moves into the range of *R*_*d*_ and *t*_*1*_ is the time when the machine moves out of the range.

#### 2.1.1 Performance index

In this study, the performance index of a karst cave can be defined as the variation in energy density at the top of the cave:

$$J(u)=\int_{t_{0}}^{t_{1}}\frac{dw_{0}}{dt}\sigma_{0}dt$$ (5)

where *w*_0_ is the vertical displacement and *σ*_0_ is the vertical stress of point (*x*_0_, *y*_0_, *z*_0_).

The displacement and stress in the elastic semi-infinite space can be solved using the Mindlin solution ‎[32, 33]. That is,

$$w_{0}(x)=\iint_{\Omega }\frac{P}{\pi r^{2}}\frac{(x-x_{0})}{16\pi G(1-\mu )}[\frac{z_{0}-z}{{R_{1}}^{3}}+\frac{\left(3-4\mu )(z_{0}-z\right)}{{R_{2}}^{3}}-\frac{6z_{0}z(z_{0}+z)}{{R_{2}}^{5}}+\frac{4(1-\mu )(1-2\mu )}{R_{2}(R_{2}+z_{0}+z)}]\mathrm{ds}$$ (6)

$$\sigma_{0}(x)=\iint_{\Omega }\frac{P}{\pi r^{2}}\frac{(x-x_{0})}{8\pi (1-\mu )}\{\frac{1+2\mu }{{R_{1}}^{3}}-\frac{\left(1-2\mu \right)}{{R_{2}}^{3}}-\frac{3(z_{0}-z{)}^{2}}{{R_{1}}^{5}}-\frac{3(3-4\mu )(z_{0}+z{)}^{2}}{{R_{2}}^{5}}+\frac{6c}{{R_{2}}^{5}}[z+(1-2\mu )(z_{0}+z)+\frac{5z_{0}(z_{0}+z{)}^{2}}{{R_{2}}^{5}}]\}ds$$ (7)

where *P* is the shield front thrust, *μ* is Poisson’s ratio, *G* is the shear modulus, and *r* is the radius of the excavation face. *Ω* is the range of the excavation face, which can be defined as

$$\Omega =\left\{(x,m,n)|\sqrt{(m-y{)}^{2}+(n-z{)}^{2}}\leq r^{2}\right\}$$ (8)

*R*_1_ and *R*_2_ are defined as

$$R_{1}=\sqrt{(x_{0}-x{)}^{2}+(y_{0}-y{)}^{2}+(z_{0}-z{)}^{2}}$$ (9)

$$R_{2}=\sqrt{(x_{0}-x{)}^{2}+(y_{0}-y{)}^{2}+(z_{0}+z{)}^{2}}$$ (10)

The relationship between the shield tunneling speed and positive thrust can be obtained by fitting by using experimental data [34].


$$\dot{x}=b_{1}e^{\frac{P}{b_{2}}}+b_{3}$$ (11)

The optimal shield tunneling speed near karst caves can be obtained by solving *u*(*t*)∈*U*_*ad*_ and *x*(*t*) to minimize the value of *J*(*u*) defined in Eq (5). That is,

$$\left\{ \begin{array}{c} \dot{x}=u(t)\\ x(t_{0})=x_{0}-R_{d},\;x(t_{1})=x_{0}+R_{d} \end{array}\right.$$ (12)

The displacement of the elastic semi-infinite space can be obtained by simplifying Eq (6) as,

$$w_{0}(x)=\frac{P}{\pi r^{2}}\frac{(x-x_{0})}{16\pi G(1-\mu )}I_{w}(x)$$ (13)

$$I_{w}(x)=\iint_{\Omega }[\frac{z_{0}-z}{{R_{1}}^{3}}+\frac{\left(3-4\mu )(z_{0}-z\right)}{{R_{2}}^{3}}-\frac{6z_{0}z(z_{0}+z)}{{R_{2}}^{5}}+\frac{4(1-\mu )(1-2\mu )}{R_{2}(R_{2}+z_{0}+z)}]ds$$ (14)

**Fig 2:**
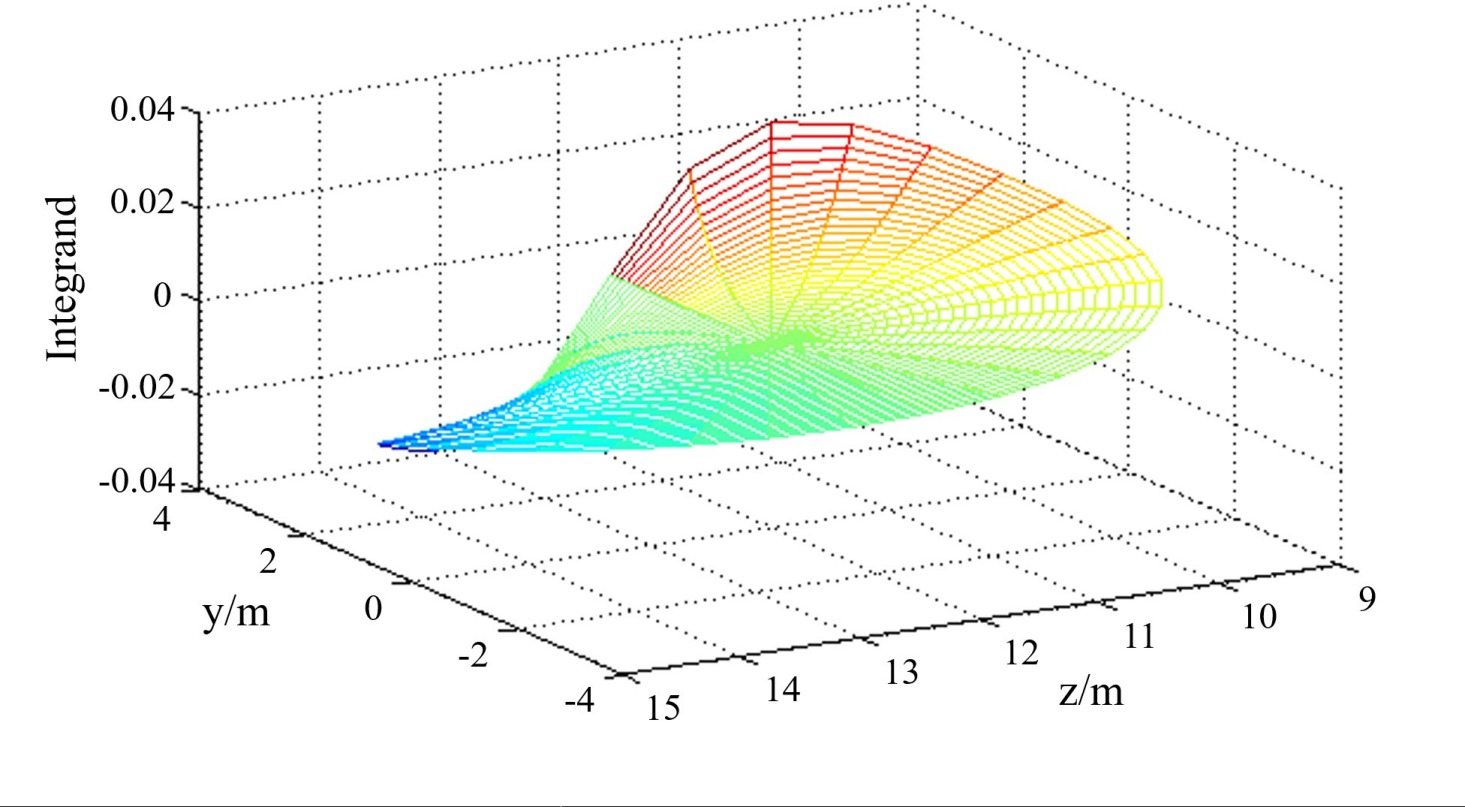
The numerical integration of *I*_*w*_.

**Fig 3:**
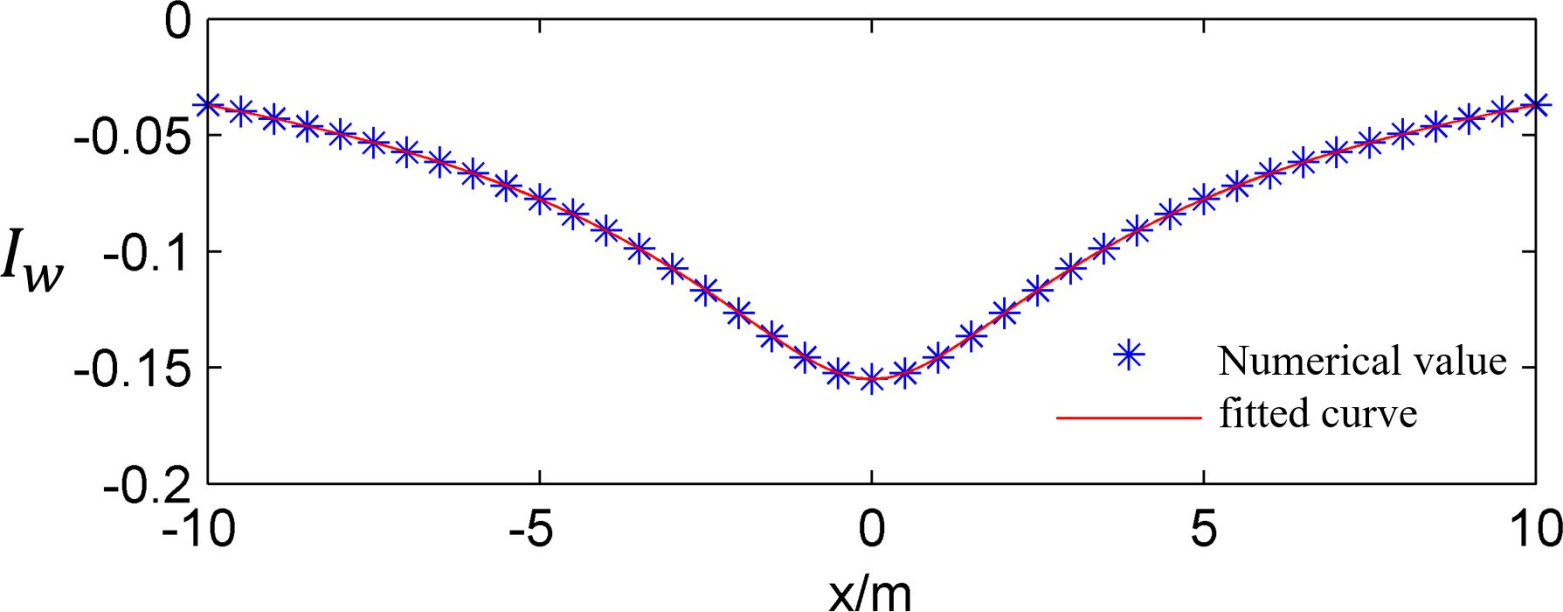
The relationship between *I*_*w*_ and *x*.

The integral region is divided into equal spacing grids according to polar coordinates, as shown in Fig 2, and the value of *I*_*w*_(*x*) on each node is calculated under the condition that the x value is constant. Then, as the x value changes, the *I*_*w*_(*x*) value is calculated, and finally, the relationship of the change in *I*_*w*_(*x*) with respect to x is fitted, as shown in Fig 3. The same method can be used to calculate the vertical stress.

#### 2.1.2 Penalty function

To ensure that the shield tunneling machine can reach the specified position at end time *t*_1_, the penalty function term *N*[*x*(*t*_1_)−(*x*_0_+*R*_*d*_)]^2^ is introduced into the performance indicator function to increase the continuity and accuracy during the solving process:

$$J(u)=\int_{t_{0}}^{t_{1}}\frac{dw_{0}}{dt}\sigma_{0}dt+N[x(t_{1})-(x_{0}+R_{d}){]}^{2}$$ (15)

#### 2.1.3 Gradient method

The time-dependent problem can be solved by defining the Hamilton function as

$$H=-\frac{dw_{0}}{dt}\sigma_{0}+\lambda u$$ (16)

where the multiplier function *λ*(*t*) is the covariant variable. The optimal control u*(*t*) of the system and the corresponding state curve x*(*t*) satisfy

$$\underset{u\in U_{\mathrm{ad}}}{\max}H(x^{*}(t),u,\lambda (t))=H(x^{*}(t),u^{*}(t),\lambda (t))$$ (17)

$$\dot{\lambda }=-\frac{\partial H}{\partial x}$$ (18)

$$H(x^{*}(t),u^{*}(t),\lambda (t))\equiv H|t_{1}^{*})=0$$ (19)

Thus, the analytical solution to the displacement and stress can be obtained by solving Eqs (16)–(18) with the initial condition (*t* = *t*_*0*_) and final condition (*t* = *t*_*1*_) using the gradient method. The specific steps are as follows:

1. Define the penalty factor *N*>0 and final state tolerance *ε*_0_>0.
2. Define the initial control function *u*_0_(*t*), allowable error *δ*_0_>0 and *K* = 0.
3. Replace *u*(*t*) with *u*_*K*_(*t*), and obtain *x*_*K*_(*t*) from Eq (5).
4. Based on *λ*_*K*_(*t*_1_) = 2*N*[*x*(*t*_1_)−(*x*_0_+*R*_*d*_)] and Eq (18), obtain *λ*_*K*_(*t*) by backward integration.
5. Calculate the gradient
$$h(u_{K}(t))=-\frac{\partial H}{\partial u}(x_{K}(t),u_{K}(t),\lambda_{K}(t))$$ (20)
6. Modify the control function
$$u_{K+1}\left(t\right)\left\{ \begin{array}{c} v_{max},u_{K}+a_{K}^{*}∙h(u_{K}(t))\geq v_{max}\\ u_{K}+a_{K}^{*}∙h(u_{K}(t)),v_{min}<u_{K}+a_{K}^{*}∙h(u_{K}(t))<v_{max}\\ v_{min},a_{K}^{*}∙h(u_{K}(t))\leq v_{max} \end{array}\right.$$ (21)
  where $a_{K}^{*}$ is the parameter for improving the efficiency of the algorithm. In addition, the following condition should be satisfied:
$$J(u_{K}+a_{K}^{*}∙h(u_{K}(t)))=\underset{a_{K}>0}{\min}J(u_{K}+a_{K}∙h(u_{K}(t)))$$ (22)
7. Calculate the error (*δ*) as follows:
$$\delta =\left|\frac{J(u_{K+1})-J(u_{K})}{J(u_{K})}\right|$$ (23)
  If *δ*<*δ*_0_, then go to Step (8). Otherwise, *K* = *K*+1, and go to Step (3).
8. Calculate the final state error (*ε*) as follows:
$$\varepsilon =|x_{K}(t_{1})-(x_{0}+R_{d})|$$ (24)
  If *ε*<*ε*_0_, the optimal shield tunneling speed (*u*_*K*_) is obtained. Otherwise, repeat the iteration by increasing the value of *N* and proceeding to Step (2).

### 2.2 Problem description

**Fig 4:**
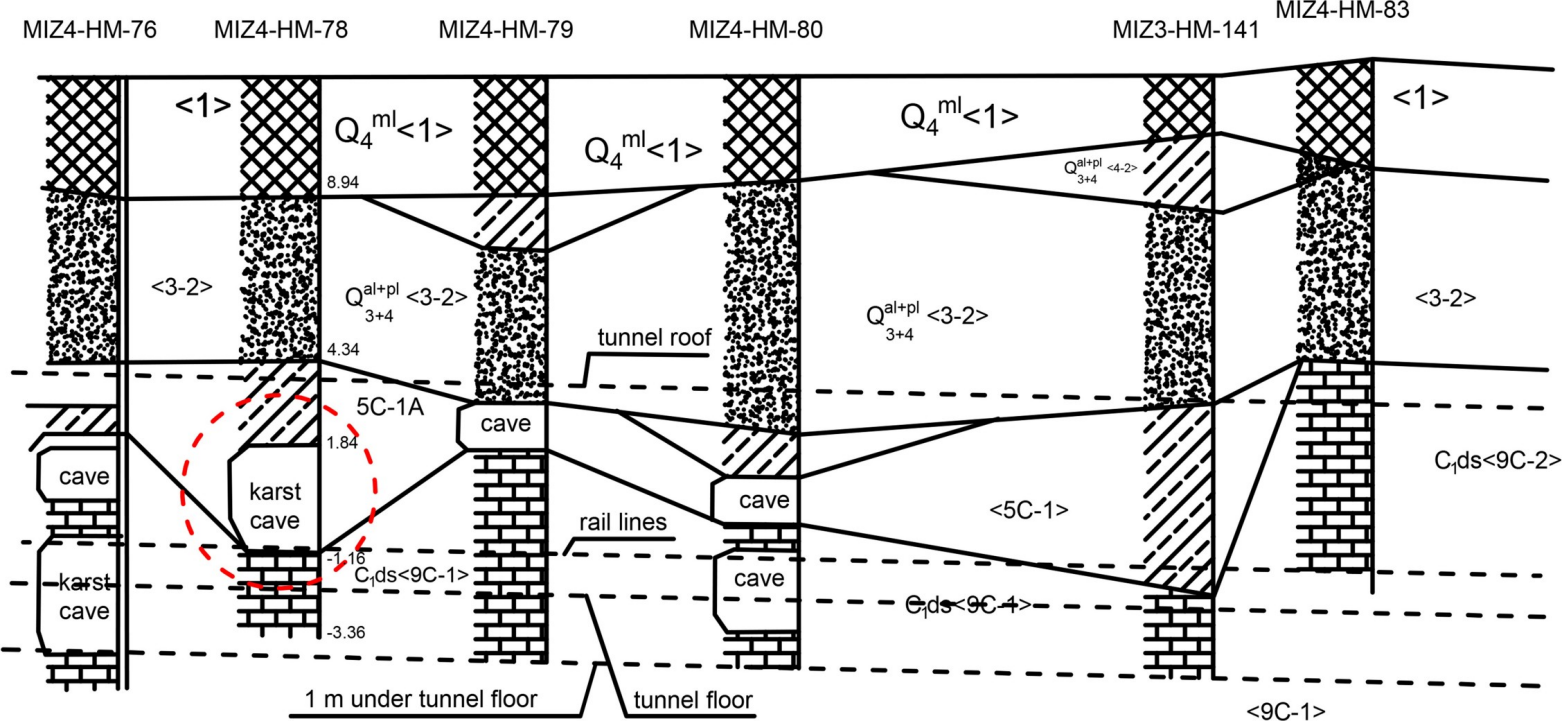
Geological details of the karst region in which the tunnel was constructed.

In this section, the developed model was implemented to study a 20-km-long subway tunnel project in a karst region of South China. The tunnel was constructed mainly through a Quaternary sand layer and limestone stratum with many karst caves, which poses significant engineering risks during tunnel construction. Therefore, it is critical importance for controlling the shield tunneling speed to ensure the stability of the adjacent karst caves. Fig 4 shows the geological details of the section investigated in this study. The length of the section is approximately 1301 m. The composition of the section from the top to the bottom includes the artificial fill layer <1>, the alluvial-diluvial medium coarse sand layer <3–2>, the alluvial-diluvial silty clay <4N-2>, the soft and plastic residual soil layer <5C-1A> and the limestone aeolian rock belt <9C-1>.

The drilling data from 422 boreholes show that there are many karst caves, most of which are not filled or partially filled. The tunnel is constructed by the shield tunneling method, mainly through the eluvial soil layer <5C-1A> and sand layer <3–2> and partially through limestone <9C-1>. The maximum burial depth of the shield tunnel is 8.6 m, and the outer diameter of the tunnel lining is 6000 mm.

This study mainly focuses on investigating the stability of a karst cave (11 m in depth) encountered in borehole MIZ4-HM-78 (Fig 4) during the shield tunneling process. The shield machine is driven along the positive direction of the *X* axis with a horizontal distance of 5 m from the karst cave and the central coordinate of the excavation surface is (*x*,0,12). The coordinates of the shallowest point of the cave are (0,5,11). The soil characteristics surrounding the karst used in this study (Table 1) were obtained from a previous relevant study [35].

**Table 1:** Details of the soil conditions surrounding the karst cave studied in this project [35].

Number	Elastic modulus (MPa)	Weight density (kN/m^3^)	Internal friction angle (kPa)	Poisson’s ratio	Lateral pressure coefficient
<5C-1A>	60.0	19.1	15	20	0.5
<3–2>	30.0	20.5	33	0.33	0.35

In addition, the stability of a karst cave is related to many factors, such as the filling of the cave, the state of the groundwater, and the density of the backfill grouting. The focus of this paper is to ensure the stability of karst caves in the process of shield construction by optimizing the tunneling speed of the shield machine.

## 3 Results and discussion

According to the successful completion of similar projects [36, 37] and previous research [1, 7, 12], the shield tunneling speed can be controlled between 10 mm/min and 30 mm/min with synchronous grouting when crossing karst caves, so the control constraint is 10 mm/min≤ u ≤ 30 mm/min.

**Fig 5:**
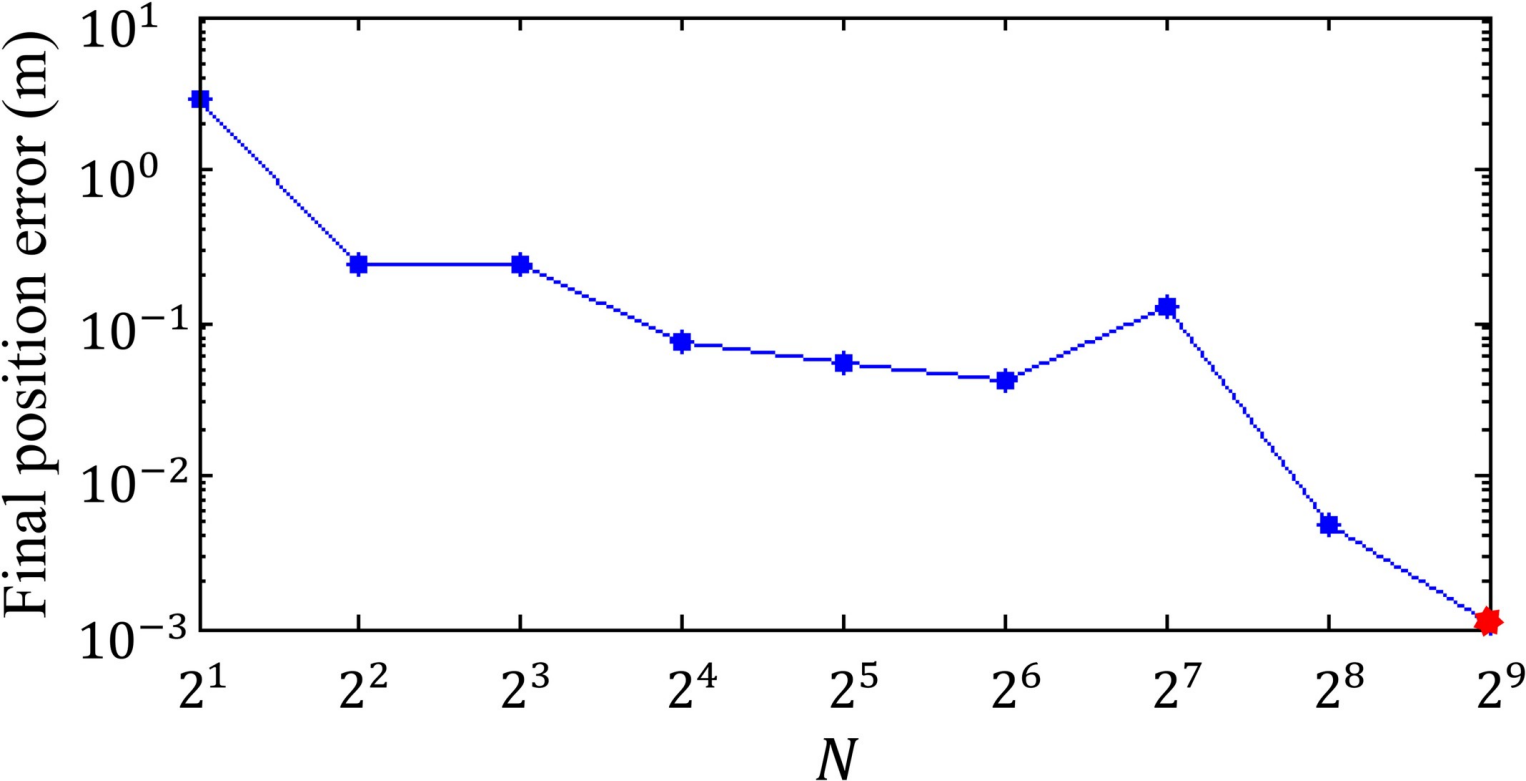
Final position error of the shield machine under different values of the penalty factor (*N*).

According to the research results of [31], when karst caves are located on both sides of the shield machine, the radius of the disturbance area is 1.5 times the shield diameter, which is 1.5* 6.0 m = 9.0 m in this project. To reduce the computational time, the symmetry of the index function is considered, *i*.*e*., the final state is *x*(*t*_1_) = 0. Assuming that the completion time of the shield tunneling process is approximately 20 hours, *t*_1_ = 600 min when half of the process is completed and the index function is $J(u)=\int_{t_{0}}^{t_{1}}\frac{dw_{0}}{dt}\sigma_{0}dt+N\cdot (x(t_{1}){)}^{2}$. The iteration process involves increasing the penalty factor *N* until a predefined final position error of the shield machine is achieved (*i*.*e*., the final state is *x*(*t*_1_) = −0.0011 when *N* = 512, as shown in Fig 5).

**Fig 6:**
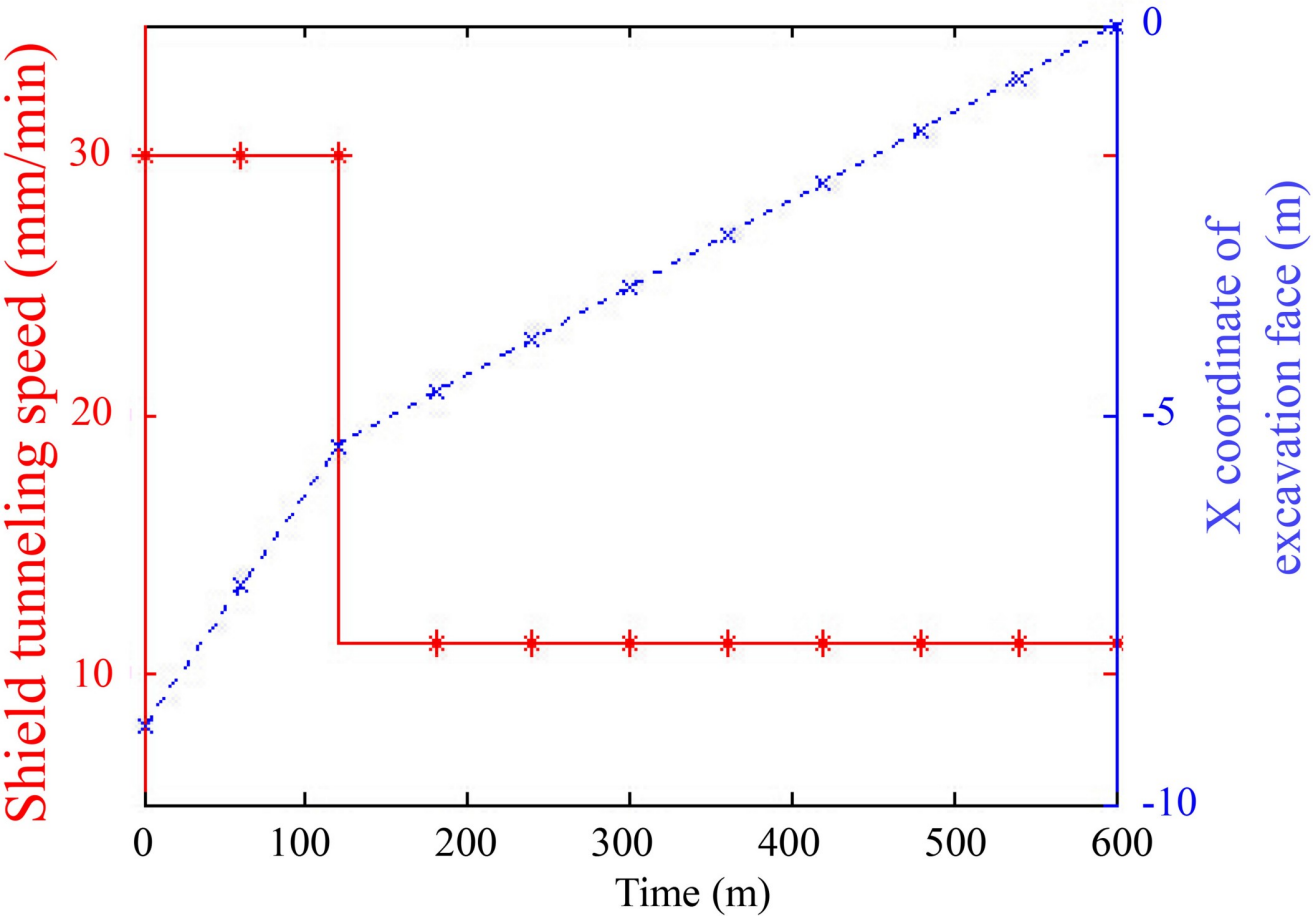
Optimal shield tunneling speed and corresponding displacement.

When *t* = *t*_1_, the optimal shield tunneling speed and the corresponding displacement are shown in Fig 6. To ensure safety during tunnel construction, the shield tunneling speed should be controlled to below 30 mm/min during the first 120 min of the simulation. When the shield machine reaches 5.4 m from the karst cave, the shield speed needs to be reduced to 11.2 mm/min to reduce the disturbance to the karst cave. According to the symmetry of the problem, the speed of 30 mm/min can be restored when the shield machine moves 5.4 m past the karst cave.

**Fig 7:**
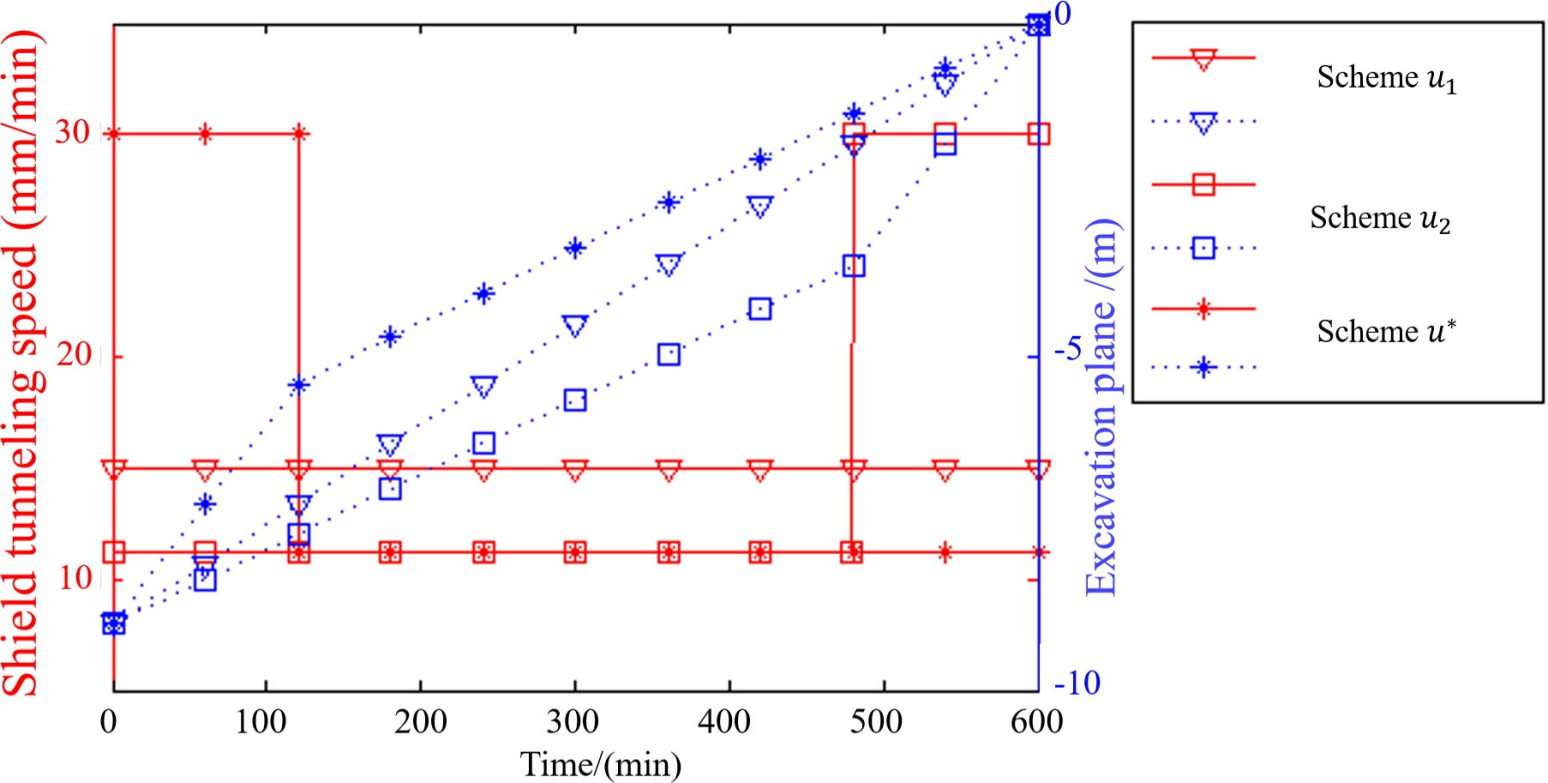
Comparison of different shield tunneling schemes.

**Fig 8:**
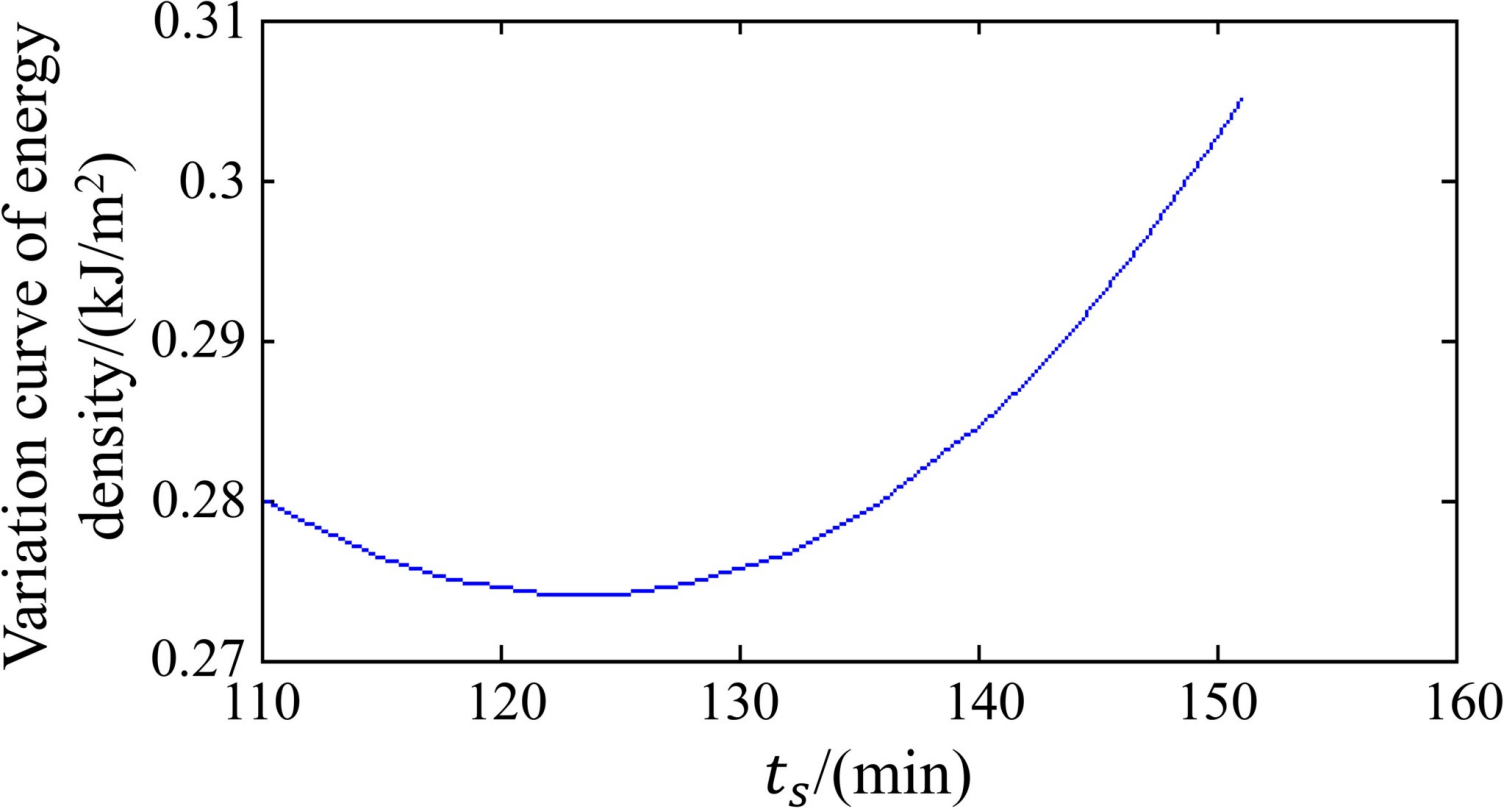
Time-dependent variation in energy density.

To identify the optimal shield tunneling strategies, three shield tunneling schemes were investigated. The details are described in Table 2, and the theoretical predictions are shown in Fig 7. Among the schemes, scheme u* (*i*.*e*., deceleration) could lead to the lowest variation in energy density at the top of the karst cave and therefore represents the best shield tunneling strategy. The time-dependent variation in energy density in Fig 8 demonstrates that for scheme u*, the minimum variation in energy density at the top of the karst cave is reached at deceleration time *t*_*s*_ = 160 min. In summary, the average shield tunneling speed outside the karst cave influence area is approximately 30 mm/min, while the average shield tunneling speed in the karst cave influence area (which extends 6 m from the karst cave boundary) is approximately 10 mm/min. These results are consistent with those of previous studies [36, 37].

**Table 2:** Details of the three shield tunneling schemes used in this study.

Shield tunneling schemes	Description	The variation in energy density at the top of the karst cave (kJ/m^2^)
u*	Deceleration	0.2741
*u*_1_	Uniform passing	0.5886
*u*_2_	Acceleration	1.4888

## 4 Conclusion

In this study, a theoretical model was developed to ensure the stability of karst caves during the shield tunneling process. The model uses an iteration process to identify the optimal shield tunneling speed, which has a minimal impact on the stability of a nearby karst cave. The developed model was implemented to investigate a subway project constructed in a karst region of South China. The following are the major findings of this work:

- To ensure safety during tunnel construction, the shield tunneling speed should be controlled to below 30 mm/min when approaching a karst cave (during the first 120 min of the simulation). When the shield machine moves into the affected zone (e.g., 5.4 m from the karst cave), the shield speed needs to be reduced to 11.2 mm/min. According to the model symmetry, the speed of 30 mm/min can be restored when the shield machine moves out of the affected zone.
- The shield deceleration strategy could lead to less variation in energy density at the top of the karst cave, and in this case, the minimum variation in energy density at the top of the karst cave is reached at deceleration time *t*_*s*_ = 160 min.
  - In the simulation, the average shield tunneling speed outside the karst cave influence area was approximately 30 mm/min, while the average shield tunneling speed in the karst cave influence area (which extends 6 m from the karst cave boundary) was approximately 10 mm/min, which is consistent with engineering experience.
- This study provides a new idea and a theoretical basis for optimizing the average shield tunneling speed, minimizing the disturbance to karst cave stability and ensuring the safety of tunnel construction in karst regions.
